# The Integrated Data Repository Toolkit (IDRT): accelerating translational research infrastructures

**DOI:** 10.1186/2043-9113-5-S1-S6

**Published:** 2015-05-22

**Authors:** Christian Bauer, Thomas Ganslandt, Benjamin Baum, Jan Christoph, Igor Engel, Matthias Löbe, Sebastian Mate, Hans-Ulrich Prokosch, Ulrich Sax, Sebastian Stäubert, Alfred Winter

**Affiliations:** 1 Department of Medical Informatics, University Medical Center Göttingen, Göttingen, Germany; 2 Medical Center for Information and Communication Technology, Erlangen University Hospital, Erlangen, Germany; 3 Chair of Medical Informatics, Friedrich-Alexander-University of Erlangen-Nuremberg, Erlangen, Germany; 4 Institute for Medical Informatics, Statistics and Epidemiology (IMISE), University Leipzig, Leipzig, Germany

## Characterization

i2b2 tool/plugin, patient recruitment, feasibility, translational research, ETL, data integration, open source, data warehouse.

## Description

**Figure 1:**
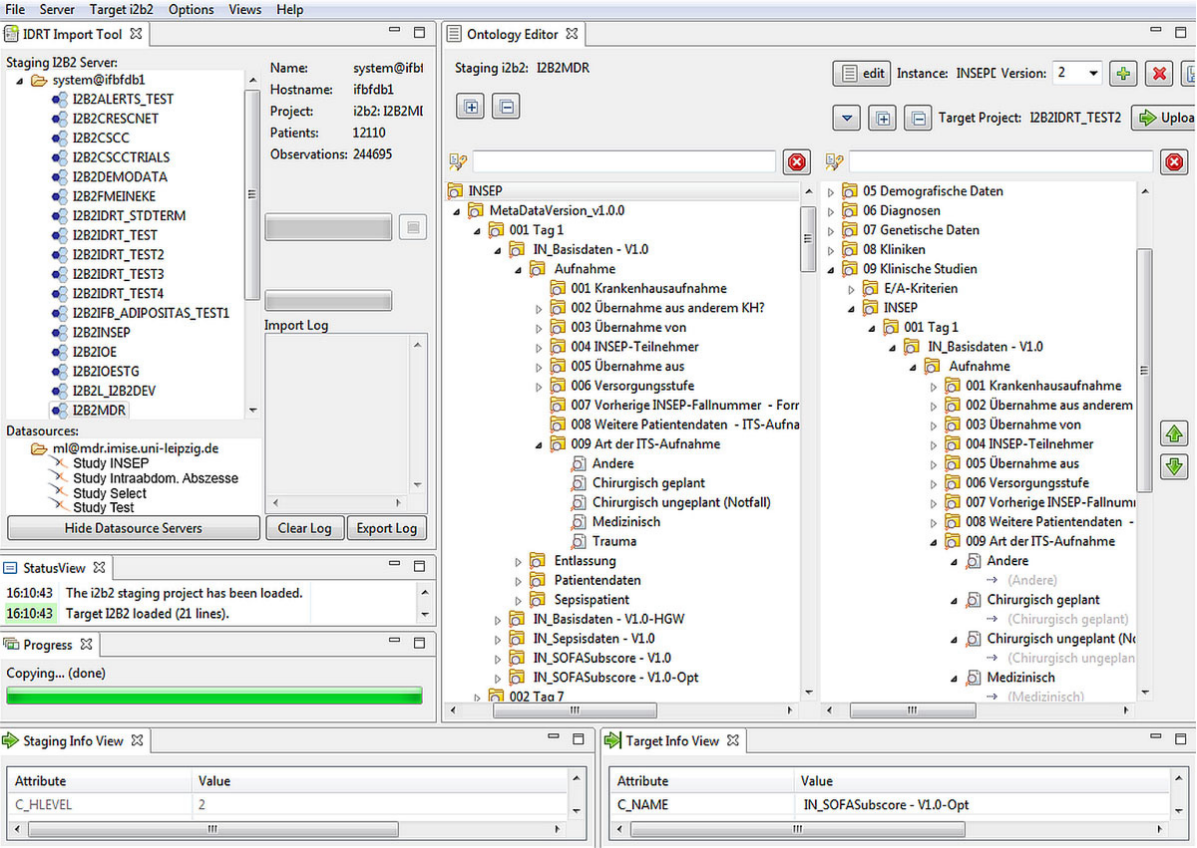
The IDRT Import Tool with the i2b2 server/project and Datasourcedata source browser (left window) and the i2b2 Ontology Editor (right side).

The Open Source software i2b2 [1] provides a translational research platform for storing biomedical data and querying these data with a user-friendly interface for researchers (Figure 1). Despite its powerful features, it is lacking user-friendly tools for installation and configuration, the import of source data and the creation of a comprehensive navigational structure (i2b2 ontology). To close these gaps, the Integrated Data Repository Toolkit (IDRT), consisting of three software tools, has been created. The i2b2 Wizard provides a shell GUI for the installation and configuration of i2b2 instances, projects and users. The i2b2 Import Tool offers a GUI for browsing i2b2 projects and importing data in various standard data formats into i2b2 (e.g., textual (CSV), relational (SQL) or structured data (CDISC ODM/XML)), as well as a dedicated extractor for biomaterial data. During import, i2b2 ontologies are automatically created from metadata included in the source data. The i2b2 Ontology Editor (IOE), being part of the i2b2 Import Tool, can be used for enhancing these i2b2 ontologies. Besides standard functions like rearranging, adding, deleting and renaming folders and items, the IOE is capable of augmenting i2b2 ontologies with more advanced i2b2 functions. By utilizing the two windows of the IOE (one showing the unaltered source i2b2 ontology and the other the manually created target i2b2 ontology), mappings can be achieved by simple drag-and-drop operations. For example, start and end dates can be added to items by dragging a date item onto a fact item. Medical terminologies can easily be imported with the IDRT (e. g. ICD-10, LOINC) and can also be mapped via the same drag-and-drop operations to data elements (expandable beyond the supplied terminologies via a regular expression editor in the IOE). The IDRT tools support the more advanced i2b2 functionalities for “fact nesting”, called “modifiers”. Since the i2b2 web browser query application (i2b2 Web Client) does not support simple access and visualization of modifiers, an IDRT plugin was created that is able to display, combine and export related facts. Additional documentation for enhanced i2b2 usage is provided on the IDRT website [2].

## Status of development

In development (November 2014).

## Users

University Medical Center Göttingen.

## Link

http://idrt.imise.uni-leipzig.de/
